# Technical and Biological Biases in Bulk Transcriptomic Data Mining for Cancer Research

**DOI:** 10.7150/jca.100922

**Published:** 2025-01-01

**Authors:** Hengrui Liu, Yiying Li, Miray Karsidag, Tiffany Tu, Panpan Wang

**Affiliations:** 1 Cancer Research Institute, Jinan University, Guangzhou, China.; 2 The First Affiliated Hospital of Jinan University, Guangzhou, China.; 3 Yinuo Biomedical., Tianjin, China.; 4 Qingdao Public Health Clinical Center, Qingdao, China.; 5 Canyon Crest Academy, San Diego, CA, USA.; 6 Hinsdale Central High School, Hinsdale, IL, USA.

## Abstract

Cancer research has been significantly advanced by the integration of transcriptomic data through high-throughput sequencing technologies like RNA sequencing (RNA-seq). This paper reviews the transformative impact of transcriptomics on understanding cancer biology, focusing on the use of extensive datasets such as The Cancer Genome Atlas (TCGA) and Genotype-Tissue Expression (GTEx). While transcriptomic data provides crucial insights into gene expression patterns and disease mechanisms, the analysis is fraught with technical and biological biases. Technical biases include issues related to microarray, RNA-seq, and nanopore sequencing methods, while biological biases arise from factors like tumor heterogeneity and sample purity. Additionally, misinterpretations often occur when correlational data is erroneously assumed to imply causality or when bulk data is misattributed to specific cell types. This review emphasizes the need for researchers to understand and mitigate these biases to ensure accurate data interpretation and reliable clinical outcomes. By addressing these challenges, the paper aims to enhance the robustness of cancer research and improve the application of transcriptomic data in developing effective therapies and diagnostic tools.

## 1. Introduction

Cancer remains one of the most complex and challenging diseases facing medical science today, characterized by abnormal cell growth with the potential to invade or spread to other parts of the body. Traditional approaches to understanding and treating cancer have often focused on histological analysis and targeted molecular studies^1^. The advent of transcriptomic data mining has opened new horizons for unraveling the molecular intricacies of cancer at a genomic level^2^. Transcriptomics, the comprehensive study of RNA transcripts produced by the genome, provides deeper insights into cellular function and regulation^3^, as well as therapeutic resistance^4^. Recent advancements in high-throughput sequencing technologies have enabled the accumulation of vast amounts of transcriptomic data, which, when effectively mined, offer profound potential to enhance our understanding of cancer biology. This data reveals crucial information about gene expression patterns specific to different types of cancers and their stages, enabling the identification of novel biomarkers and therapeutic targets. However, transcriptomic analysis alone has its limitations, as it primarily focuses on RNA expression and may not fully capture post-translational modifications or metabolic processes that drive cancer progression. To overcome these limitations, integrating multi-omics approaches, such as proteomics and metabolomics, has become increasingly important^5^, ^6^. Proteomics allows for the examination of protein expression and modifications, while metabolomics provides insights into the biochemical changes within cancer cells^7^. By combining these datasets with transcriptomic analysis, researchers can gain a more holistic understanding of cancer biology, potentially leading to more accurate biomarker discovery and therapeutic targeting.

Transcriptomics, which involves the study of RNA transcripts derived from DNA, is often considered technically easier than direct DNA detection for several reasons: 1) Amplification Simplicity: RNA, particularly mRNA, is generally more abundant in cells compared to specific sequences of DNA that might be of interest, such as those associated with mutations. This abundance allows for easier sampling and detection without the need for extensive amplification. In contrast, DNA detection often requires precise amplification of specific regions, which can be technically challenging and prone to errors. 2) Dynamic Range of Expression: RNA offers a dynamic range of expression levels, which can provide more detailed information about cellular processes and states than DNA. This dynamic information is crucial for understanding disease states like cancer, where gene expression can change dramatically and inform about the severity and progression of the disease. 3)Technical Tools and Protocols: The tools and protocols for RNA analysis, such as RNA-Seq, have become highly standardized and sensitive, allowing for a broad analysis of the transcriptome with relative ease. Technologies for detecting and quantifying RNA are robust, well-understood, and can simultaneously measure the expression levels of thousands of genes. This contrasts with certain DNA detection techniques, which might require more intricate setups to identify mutations or epigenetic changes. 4) Less Complexity in Analysis: RNA transcripts are less complex than whole genomes because they represent a subset of the genomic content that is actively expressed in cells. Analyzing RNA avoids many complexities associated with DNA analysis, such as repetitive sequences and structural variations, which can complicate the mapping and interpretation of DNA sequencing data. 5) Temporal and Spatial Resolution: Given that mRNA is generally much more abundant in cells, transcriptomics can capture temporal and spatial differences in gene expression, providing insights into how genes are regulated over time and across different tissues or in response to various stimuli. This aspect of RNA study can be more directly and easily linked to phenotypic changes than DNA-based studies, which typically provide a static and broad view of genetic potential without context. 6) Cost-Effectiveness: Generally, RNA sequencing technologies have become more cost-effective than some forms of DNA sequencing, especially when considering the level of detailed information they can provide regarding the active biological processes within a cell or tissue.

A number of open transcriptomic data are now available for cancer studies, such as The Cancer Genome Atlas (TCGA)^8^ and Genotype-Tissue Expression (GTEx)^9^, ^10^, two of the most commonly used databases for data mining. The TCGA and GTEx projects are two significant research initiatives that have provided the scientific community with extensive genomic and transcriptomic data. These datasets are foundational for understanding cancer biology and normal tissue function, respectively. TCGA is a landmark cancer genomics program, which molecularly characterized over 20,000 primary cancers and matched normal samples spanning 33 cancer types. This project was launched jointly by the National Cancer Institute (NCI) and the National Human Genome Research Institute (NHGRI). GTEx, on the other hand, was established to create a reference database and tissue bank to study the relationship between genetic variation and gene expression in various non-diseased human tissues. Together, TCGA and GTEx provide complementary datasets where TCGA focuses on cancerous tissues and GTEx on normal tissues across many of the same tissue types. Researchers can use these datasets to: 1) Compare normal and cancerous tissues: Understanding differences in gene expression and genetic mutations between normal and tumor tissues can help pinpoint cancer-specific therapeutic targets. 2) Enhance biomarker discovery: By comparing genetic expressions across healthy and diseased tissues, scientists can identify specific markers responsible for disease. 3) Improve our understanding of disease mechanisms: Analyzing how genes are regulated differently in healthy versus diseased states offers insights into the pathways and mechanisms underlying various conditions, including cancers.

In recent years, there has been a marked increase in academic studies focused on mining open data, leading to diverse approaches in their structure and methodology. Early research often concentrated on investigating single genes in specific cancer types^11^-^15^, offering relatively focused insights. However, with the advent of advanced data mining techniques, researchers can now process much larger datasets with greater efficiency. This has paved the way for pan-cancer studies of single genes^16^, ^17^, representing a significant expansion in scope. Similarly, there have been studies examining sets of genes, such as those within a gene family^18^ or biological pathway^19^-^21^, for a single cancer type, often incorporating survival models. These studies, while broader, are still not the most complex examples of multi-dimensional research. When considering cancer types as one dimension and genes as another, the study of a single gene in a single cancer type can be viewed as zero-dimensional. In contrast, research on single genes across multiple cancer types (pan-cancer studies) or gene sets within a single cancer type are one-dimensional approaches. There are also examples of two-dimensional studies, such as research involving gene sets across multiple cancer types (pan-cancer gene set studies)^22^-^29^. Looking ahead, it is anticipated that data mining will enable even more complex, multi-dimensional studies, such as those involving pan-cancer gene sets analyzed over timelines^30^ or within single-cell^31^/spatial contexts^32^, ^33^.

Data mining papers primarily concentrate on several key aspects: 1) Understanding Gene Expression Patterns: Transcriptomic data provides insights into the RNA expression levels across different tissues, conditions, or stages of a disease. Mining these data helps researchers identify specific genes that are upregulated or downregulated under various circumstances, which can elucidate the biological pathways and mechanisms involved in health and disease. 2) Disease Diagnosis and Prognosis: By analyzing gene expression profiles from patient samples, scientists can develop biomarkers for diagnosing diseases or predicting outcomes. For instance, certain cancers exhibit unique expression signatures that can be used not only for diagnosis but also to predict patient response to treatments, enabling more personalized medicine approaches. 3) Drug Discovery and Repurposing: Transcriptomic data can reveal how genes respond to different drugs, providing insights into their mechanisms of action or potential side effects. This information is crucial for drug development and repurposing existing drugs to treat new conditions. In addition, in many previous studies^34^, ^35^, transcriptomic studies provide a comprehensive view of the molecular changes associated with drug resistance, enabling researchers and clinicians to develop more effective strategies for overcoming resistance and improving cancer treatment outcomes. 4) Understanding Complex Gene Networks: Data mining allows scientists to build networks of gene interactions and regulatory mechanisms. Understanding these networks can help in pinpointing key regulatory genes or transcription factors that could be potential targets for therapeutic intervention, such as network pharmacological studies^36^, ^37^. 5) Cross-Disease Analysis: Open transcriptomic databases often contain data from a variety of diseases and conditions. Researchers can use this data to perform cross-disease analyses, identifying common genes or pathways that might be involved in multiple diseases, such as ferroptosis^38^, ^39^. This can help in understanding the shared genetic basis of related conditions. Notably, open databases also offer resources for the increasingly popular Mendelian randomization analysis, which is used for cross-disease association studies^40^, ^41^. 6) Evolutionary and Comparative Studies: Comparing transcriptomic data across different species can provide insights into the evolution of gene expression and regulation. Such studies help in identifying conserved genes and pathways, underscoring their fundamental biological importance.

There are some advantages of utilizing open data mining. Open data promotes collaboration among scientists worldwide, enabling larger-scale analyses and validation of findings across multiple datasets. This collaborative environment enhances the reliability and reproducibility of scientific research, as findings can be tested and verified independently by other groups. Access to open transcriptomic data allows researchers to conduct high-level research without the need for expensive and time-consuming data generation. This is particularly beneficial for smaller labs or institutions with limited resources. Open data serves as an excellent resource for training the next generation of scientists. Students and early-career researchers can learn data analysis techniques and explore real-world biological data sets, gaining valuable experience. Data mining of open transcriptomic data is an essential endeavor in the era of big data biology. It accelerates scientific discovery, enhances understanding of molecular biology, and fosters the development of new therapeutic strategies. By leveraging these vast datasets, the scientific community can make significant advances in understanding and treating diseases, ultimately contributing to improved health outcomes.

**Figure 1:**
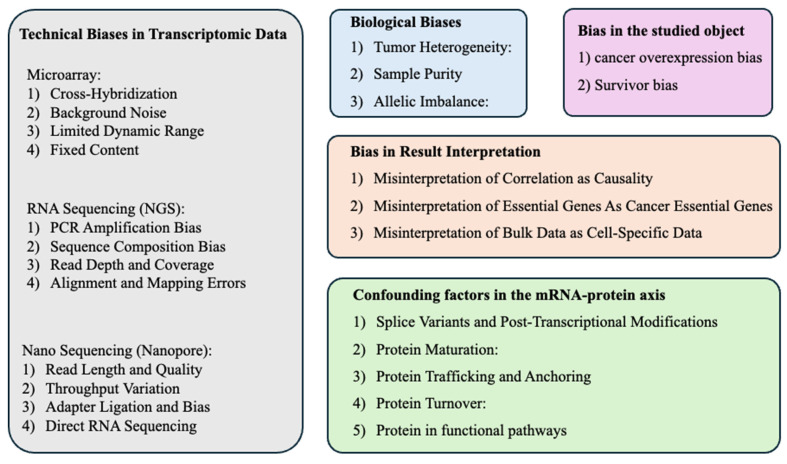
Technical and biological biases in bulk transcriptomic data mining for cancer research.

A recent review article has discussed some biases in data analysis with a more general context^42^. In this review article, we proposed a few critical considerations for the scientific community to understand the potential biases and limitations in data mining of open transcriptome data, as summarized in **Figure 1**. Hopefully, this paper can benefit the field by reminding researchers to mitigate these biases and limitations as much as possible.

## 2. Technical Biases in Transcriptomic Data

Microarray, RNA sequencing (RNA-seq) via next-generation sequencing (NGS), and nano sequencing are prominent methods for analyzing gene expression, but each comes with specific technical biases. Understanding these biases is crucial for accurate data interpretation and experimental design. Here, we briefly summarized some of the technical biases associated with each method.

Microarray^43^, ^44^: 1) Cross-Hybridization: Similar sequences can hybridize to the wrong probes, leading to false signal detection or signal interference. 2) Background Noise: Non-specific binding of labeled cDNA to the array surface can cause elevated background signals that may obscure true gene expression levels. 3) Limited Dynamic Range: The fluorescence signal is often compressed both at the low and high ends, limiting the ability to detect very low or very high expression levels accurately. 4) Fixed Content: Microarrays contain a predetermined set of probes, which limits the ability to detect novel transcripts or variants not included in the array.

RNA Sequencing (NGS)^45^-^47^: 1) PCR Amplification Bias: Amplification steps in library preparation can preferentially enrich for certain sequences, skewing the representation of RNA species. 2) Sequence Composition Bias: GC-rich or AT-rich regions can be underrepresented due to biases in fragmentation and sequencing. 3) Read Depth and Coverage: The depth of sequencing and the uniformity of coverage can affect the detection of lowly expressed genes and the quantification accuracy across the transcriptome. 4) Alignment and Mapping Errors: Misalignment of short reads, especially in regions with high sequence similarity or structural variants, can lead to incorrect transcript assembly or gene expression estimation.

Nano Sequencing (Nanopore)^48^-^50^: 1) Read Length and Quality: While capable of producing long reads, nanopore sequencing can suffer from higher error rates compared to short-read sequencing technologies, particularly in homopolymeric regions. 2) Throughput Variation: The throughput of nanopore devices can vary, influencing the consistency of data across runs. 3) Adapter Ligation and Bias: The process of adapter ligation necessary for sequencing can introduce biases, affecting the sequencing of certain types of RNA or regions. 4) Direct RNA Sequencing: Direct RNA sequencing skips the reverse transcription step, which can introduce its own biases, but this approach may also omit certain RNA modifications unless specifically accounted for.

Each of these biases necessitates specific strategies for data processing and analysis. Common bioinformatics tools, such as limma for microarray data or DESeq2 and edgeR for RNA-seq^51^, include robust normalization procedures to adjust for sequencing depth, batch effects, and amplification biases. Additionally, batch effects, which are common across platforms, can obscure true biological signals, especially when analyzing heterogeneous cancer samples. Tools like Combat (part of the sva package) and removeBatchEffect (in limma) are commonly used to correct batch effects, ensuring that the observed variations are biologically meaningful and not artifacts of the experimental setup. Awareness of these biases and the proper application of bioinformatics tools are essential for ensuring the reliability and reproducibility of studies using these technologies.

## 3. Biological Biases

In the experiment, there are also biases from biology factors: 1) Tumor Heterogeneity: Cancer is characterized by high intratumoral heterogeneity, with subpopulations of cells exhibiting distinct transcriptomic profiles. Bulk RNA-seq, which averages signals across all cells in a sample, may fail to capture this diversity, leading to biased interpretations of the tumor's molecular landscape. We will discuss this in the result interpretation section in more detail. 2) Sample Purity: The presence of non-cancerous cells, such as immune or stromal cells, in a tumor sample can confound RNA-seq results. These contaminating cells contribute their own transcriptomes, which can obscure the signals from cancer cells, particularly in studies aimed at identifying tumor-specific expression patterns. We will discuss this in the result interpretation section in more detail as well. 3) Allelic Imbalance: In cancer, there is often an imbalance in the expression of alleles due to copy number variations, loss of heterozygosity, or epigenetic modifications. RNA-seq analysis may not adequately account for this imbalance, leading to misinterpretation of gene expression data and overlooking critical oncogenic drivers.

## 4. Bias in the studied object

In cancer research, scientists frequently focus on molecules that are highly expressed, while those that are less expressed tend to be neglected despite their possible importance. For example, a gene that shows high expression in tumor samples is typically presumed to be an oncogene, leading to investigations into its potential role in advancing tumor growth. Such genes are expected to display characteristics like enhanced proliferation, invasiveness, migration, and resistance to apoptosis. Nonetheless, perspectives on this topic can differ based on various considerations that should be taken into account when formulating a hypothesis. Similarly, genes that are associated with increased risk receive more attention than those associated with protection.

The “Survivor bias” in sample collection is recently mentioned by Djamgoz and Levin^52^. They suggested that metastasis significantly impacts patient survival, and this can introduce sampling bias in tumors. For instance, tumor samples are often collected from less mobile, localized cells, while more aggressive, migrating cells may be missed if they are in transit or have formed secondary tumors that are not yet detectable or confirmed. This can lead to a misleading association between higher gene expression and better survival outcomes.

## 5. Bias in Result Interpretation

Bias in result interpretation of transcriptome data is a critical concern in genomics and can significantly impact the conclusions drawn from research, as well as their applications in clinical settings. Our recent mini-review article^42^ indicated a few challenges and complexity in genetic expression studies; here, we discuss three misinterpretations when doing transcriptomic data mining in more detail: 1) the misinterpretation of correlation as causality, 2) the misinterpretation of essential genes as cancer essential genes, and 3) the misinterpretation of bulk data as cell-specific data.

### 5.1 Misinterpretation of Correlation as Causality

Correlation analysis refers to the statistical association between the levels of gene expression or other data observed in the datasets. For example, two genes may consistently show high expression levels in a particular type of cancer tissue, suggesting a correlation. However, causality implies that one event (such as the expression of a specific gene) directly affects the occurrence of another event (such as the activation of cancer-related pathways).

Numerous studies utilize high-throughput methods to explore correlations between genes and disease phenotypes, aiming to predict the roles of genes in these conditions^20^, ^40^. While such correlations can identify potential biomarkers^13^, ^16^, ^18^, ^19^, ^23^, ^53^, ^54^, they do not confirm a gene's functional causality in a disease. This heightened expression of target genes might result partly (or entirely) from the intense expression of upstream functional proteins, leading to a correlation with the disease. However, this does not necessarily imply a significant role in disease progression. The biological network is complex; minor changes can have widespread effects. Nonetheless, if the causality is very weak and reliant on multiple coincidental factors, it should not be regarded as valid biological causality. A significant issue in interpreting RNA-seq results is the assumption that correlation implies causality. Just because two genes are expressed simultaneously in a tumor does not mean that one gene's expression causes the other or that either directly drives the cancer's progression.

Such assumptions can lead to misleading conclusions about gene functions and interactions. As an example of how disease can lead to changes in gene expression, studies on CD4+ T cell activation have shown significant alterations in gene expression during the activation process^55^, illustrating how certain diseases, particularly those involving immune responses, can result in abnormal gene expression influenced by the immune microenvironment. On the other hand, gene expression can contribute to disease phenotypes through specific genes known to enhance these conditions. For example, TRPM7 is known to encourage cancer cell proliferation; research indicates that either eliminating this gene or inhibiting it can decrease the proliferation of cancer cells^17^, ^56^, ^57^.

The distinction between "tumor driver genes" and "oncogenes" underscores the complex and often misunderstood relationship between gene expression levels and their roles in cancer progression. The confusion often arises from misinterpreting correlation as causality, which can lead to incorrect assumptions about how certain genes function in cancer. Tumor-driver genes are specifically those that contribute to the transformation of normal cells into cancerous ones by driving key changes in cellular processes like the cell cycle, growth, and DNA replication. Their role is often directly linked to the initiation and early development of cancer. For instance, mutations in genes like KRAS, PTEN, and TP53 are associated with various types of carcinogenesis^58^-^60^. TP53, when mutated, loses its tumor-suppressing capabilities, thereby allowing cells to proliferate uncontrollably. On the other hand, oncogenes may not necessarily drive the cancer but can support the tumor's survival and progression. For example, PD-1/PD-L1, expressed on immune cells, is highly expressed in various types of cancers^61^. Tumor cells express PD-L1, which binds to PD-1 on immune cells, thereby inhibiting the activity of immune cells and aiding tumor cells in evading immune system attacks^61^. The expression of PD-L1 on tumor cells undoubtedly contributes to immune escape and exacerbates malignant phenotypes^61^. Thus, while their presence exacerbates the disease, PD-1/PD-L1 should not be conflated with genes that drive the formation of tumors.

Misinterpretation occurs when high expression levels of such genes in tumors are taken as evidence of their role in tumor promotion or initiation. This misunderstanding can be problematic in therapeutic strategies, where targeting highly expressed genes does not always equate to targeting the most causally relevant factors in cancer. Many genes may show elevated expression as a consequence of the tumor environment or as part of the body's response to cancer, not necessarily as a causative factor in cancer itself. This distinction is crucial for understanding cancer's multifaceted nature, where not every highly expressed gene in a tumor is a tumor driver, and not all tumor drivers consistently show high expression levels throughout the course of the disease. Instead, the dynamic evolution of cancer involves a complex interplay of multiple genes, where the critical roles may shift as the disease progresses. Thus, effective cancer research and treatment require a nuanced understanding of gene roles beyond mere expression levels, focusing on functional impacts and causal relationships within the cancerous state.

Misinterpreting correlation as causality can lead to several issues in scientific research. Resources may be directed toward studying genes or pathways that are correlated with disease outcomes but are not causative. This misallocation can slow progress by focusing attention on less relevant biological mechanisms. Biological models built on incorrect assumptions about gene interactions and functions can lead to ineffective or suboptimal experimental designs, which in turn produce more confusing or contradictory data. Studies based on flawed interpretations are often difficult to replicate, undermining confidence in scientific findings and wasting resources on attempts to reproduce erroneous results. In clinical settings, the stakes are even higher, as patients' treatments and outcomes can be directly affected: Misinterpreting which genes are causally linked to disease can lead to the development of targeted therapies that are ineffective, which not only wastes development resources but also delays the advancement of more effective treatments. Incorrect assumptions about causality in gene expression can lead to the identification of poor biomarkers for disease diagnosis, prognosis, or treatment response monitoring, potentially leading to suboptimal patient management. Moreover, precision medicine relies heavily on accurate gene expression profiling to tailor treatments to individual patients. Errors in understanding the causal relationships between gene expressions and disease phenotypes can result in ineffective or inappropriate treatment plans.

### 5.2 Misinterpretation of Essential Genes As Cancer Essential Genes

The distinction between essential genes and tumor drivers highlights a nuanced aspect of cancer biology explored through advanced techniques like CRISPR screening. Essential genes are defined by their fundamental role in maintaining cell viability and adaptability. Their knockdown or knockout can result in the suppression of malignant phenotypes or even cell death, but this alone does not categorize them as oncogenes. This differentiation is crucial because, while tumor-driver genes are directly involved in the onset and progression of cancer, essential genes are vital for basic cellular functions across both cancerous and normal cells.

For instance, RAD51, known for its role in DNA repair, becomes crucial in the context of cancer, where its expression might be upregulated in response to the heightened DNA repair needs of rapidly dividing tumor cells. Knocking out RAD51 can effectively kill tumor cells by crippling their ability to repair DNA^62^, ^63^, thus suppressing tumor growth. Although data suggested it is associated with tumors^16^, this gene is just as vital in normal cells for the same DNA repair processes. The mere fact that its inhibition kills cancer cells does not inherently make RAD51 an oncogene. Instead, it remains categorized as an essential gene crucial for the survival and maintenance of both cancerous and normal cells.

This distinction is important in therapeutic contexts, as targeting essential genes might offer a way to attack cancer cells but also poses significant risks of harming normal cells. Understanding the difference between oncogenes, which drive cancer, and essential genes, which are vital for normal cellular functions, helps in designing targeted therapies that minimize collateral damage to healthy tissues.

### 5.3 Misinterpretation of Bulk Data as Cell-Specific Data

Many studies explore the role of differentially expressed genes between cancer and normal tissues in cancer cells from bulk sample analysis^11^, ^12^, ^24^, ^37^, ^64^-^66^. However, the reliance on bulk sample analysis to study gene expression differences between cancerous and normal tissues can lead to significant misinterpretations, particularly concerning the cellular specificity of gene expression. While bulk sequencing has been instrumental in advancing our understanding of cancer biology, it often captures a composite picture of gene expression from all cells present in a sample, not just tumor cells. This generalization can mask the cellular complexities within the tumor microenvironment (TME), which includes a diverse array of cell types, such as immune cells, fibroblasts, and endothelial cells, alongside the actual tumor cells^13^, ^16^-^20^, ^23^, ^53^, ^54^, ^67^.

Current deconvolution calculations, such as TIMER, EPIC, MCPCOUNTER, QUANTISEQ, CIBERSORT, and XCELL, are essential tools in the field of immunology and oncology for dissecting complex tissue compositions from bulk RNA sequencing data. Each tool employs unique algorithms to estimate the proportion of various cell types within a mixed cell population, facilitating a deeper understanding of the tumor microenvironment and immune landscape. However, these tools rely on pre-existing knowledge of marker genes for known cell types, which may not account for unknown cell types or variations in marker gene expression across different tissues. This reliance can lead to gaps in detection and potentially mislead researchers if marker gene profiles are incomplete or tissue-specific differences are not considered. For instance, genes found to be highly expressed in a bulk sample might primarily be expressed in B cells or T cells rather than in tumor cells themselves^13^, ^16^-^20^, ^23^, ^53^, ^54^. This can lead researchers to erroneously attribute certain gene expressions to tumor cells when, in fact, they are predominant in other cell types within the TME. Similarly, differences in ion channel expression in glioma samples of varying severities could be misleading if not contextualized by cell type^68^-^70^. Lower ion channel expression in more severe gliomas might not reflect a direct property of the tumor cells but rather a reduced presence of neurons, which naturally exhibit higher ion channel expression, within these samples^68^-^70^.

The advent of single-cell sequencing has greatly enhanced our ability to dissect these complexities by allowing for the examination of gene expression at the individual cell level. This technology reveals the heterogeneity of cell populations within tumors, providing a clearer picture of which genes are active in tumor cells versus other cell types^13^, ^16^-^20^, ^23^, ^53^, ^54^. However, even single-cell sequencing has its limitations, such as biases introduced by the prevalence of certain cell types in samples and the technical challenges of capturing every cell type efficiently. Spatial transcriptomics enhances our understanding by not only identifying which cells express specific genes but also mapping their locations within the tissue. This technique reveals the spatial organization of cell types and their gene expression patterns within the tumor microenvironment (TME), shedding light on how interactions between different cell populations may influence disease progression and therapeutic responses. Both single-cell and spatial transcriptomics are limited in their ability to detect only highly expressed genes, potentially missing information on low-expressed genes. Increasing sequencing depth can provide more information, but this significantly raises costs. Recent advances in hybrid capture approaches offer a potential solution for researchers focusing on low-expressed genes. However, these methods require pre-designed probes for hybrid capture before sequencing and may not be useful for post-sequencing data miners interested in specific low-expressed genes.

### 5.4 Confounding factors in the mRNA-protein axis

**Figure 2:**
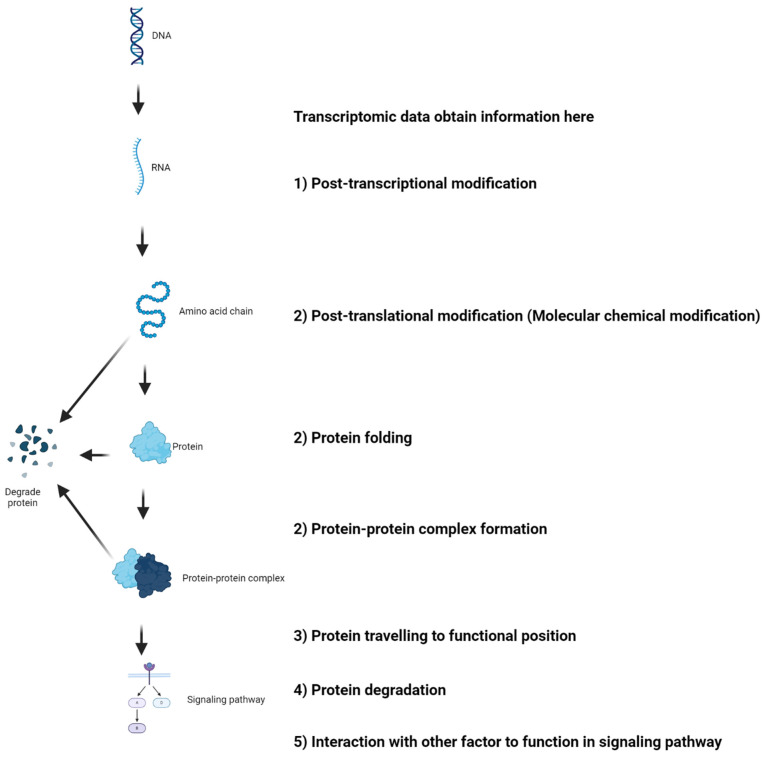
The mechanisms underlying mRNA levels do not always reflect protein abundance and functionality. This figure is adapted from the recent commentary by Djamgoz and Levin^52^ and made using the BioRender.

The recent commentary by Djamgoz and Levin^52^ on our bioinformatics-based review article^71^ addressing the role of voltage-gated sodium channels (VGSCs), particularly Nav1.5, in breast cancer raises important points regarding the potential discrepancies between transcriptomic data mining from TCGA and experimental results from in vivo and in vitro studies. Djamgoz and Levin noted that while TCGA analysis suggests a positive correlation between Nav1.5 expression and patient survival, indicating a protective prognostic factor, experimental evidence contradicts this, showing Nav1.5's association with cancer promotion^52^. They provide insightful explanations for these divergences, which merit careful consideration by researchers using bioinformatics approaches. It is crucial to recognize that mRNA levels may not always reflect protein abundance and protein function due to many mechanisms. Here, we expand on this discussion with additional considerations (**Figure 2**): 1) Splice Variants and Post-Transcriptional Modifications: The expression of Nav1.5 in cancer is mainly neonatal splice variants, which are regulated by alternative splicing and post-transcriptional modifications. 2) Protein Maturation: As an ion channel, Nav1.5 must undergo specific post-translational modifications to attain functionality. These processes include chemical modification of protein molecules, protein folding, protein-protein complex formation, etc. These modifications affect how the protein behaves and its role in cellular processes, including tumorigenesis. 3) Protein Trafficking and Anchoring: Nav1.5 must be correctly trafficked and anchored to the cellular membrane to function effectively. Any disruptions in this pathway could alter its physiological role and impact cancer cell behavior. 4) Protein Turnover: The turnover rate of the protein, including its removal from the membrane and subsequent degradation, is another layer of complexity that can influence the functional outcome and its relationship with cancer progression. 5) Protein in functional pathways: Eventually, looking at single gene expression is not recommended as protein has to function in pathways that involve many other gene expressions.

## 6. Summary and Conclusion

Cancer research has increasingly leveraged transcriptomic data thanks to advancements in high-throughput technologies. Transcriptomics offers detailed insights into gene expression and regulation, enhancing our understanding of cancer biology. Open Transcriptomics datasets have become invaluable resources for studying cancer. Despite these advancements, transcriptomic data analysis faces significant challenges due to technical and biological biases. Technical biases include issues related to microarray, RNA-seq, and nanopore sequencing technologies, while biological biases stem from factors like tumor heterogeneity and sample purity. Additionally, misinterpretations can occur when assuming correlations imply causality or when bulk data is mistaken for cell-specific data. Understanding these biases and their implications is crucial for designing robust studies and translating data into meaningful clinical outcomes. This review underscores the importance of recognizing and addressing biases in transcriptomic data mining to enhance the reliability and impact of cancer research. By highlighting specific technical and biological biases, as well as common misinterpretations, the paper aims to guide researchers in designing more accurate and informative studies. Effective mitigation of these biases is essential for advancing our understanding of cancer and developing targeted therapies. The insights provided should assist the scientific community in navigating the complexities of transcriptomic data and improving the translation of research findings into clinical applications, ultimately contributing to better cancer diagnosis, treatment, and prognosis.
